# pH Dependence of the Fluorescence Lifetime of FAD in Solution and in Cells

**DOI:** 10.3390/ijms14011952

**Published:** 2013-01-18

**Authors:** Md. Serajul Islam, Masato Honma, Takakazu Nakabayashi, Masataka Kinjo, Nobuhiro Ohta

**Affiliations:** 1 Graduate School of Environmental Science, Hokkaido University, Sapporo 060-0810, Japan; E-Mails: seraj08@yahoo.com (M.S.I.); s76113140@es.hokudai.ac.jp (M.H.); 2 Research Institute for Electronic Science (RIES), Hokkaido University, Sapporo 001-0020, Japan; 3 Faculty of Advanced Life Science, Hokkaido University, Sapporo 001-0021, Japan; E-Mail: kinjo@sci.hokudai.ac.jp

**Keywords:** FAD, autofluorescence, living cell, fluorescence lifetime imaging, intracellular pH, fluorescence decay

## Abstract

We have studied physiological parameters in a living cell using fluorescence lifetime imaging of endogenous chromophores. In this study, pH dependence of the fluorescence lifetime of flavin adenine dinucleotide (FAD), that is a significant cofactor exhibiting autofluorescence, has been investigated in buffer solution and in cells. The fluorescence lifetime of FAD remained unchanged with pH 5 to 9 in solution. However, the fluorescence lifetime in HeLa cells was found to decrease with increasing intracellular pH, suggesting that pH in a single cell can be estimated from the fluorescence lifetime imaging of FAD without adding exogenous fluorescent probes.

## 1. Introduction

**Figure 1:**
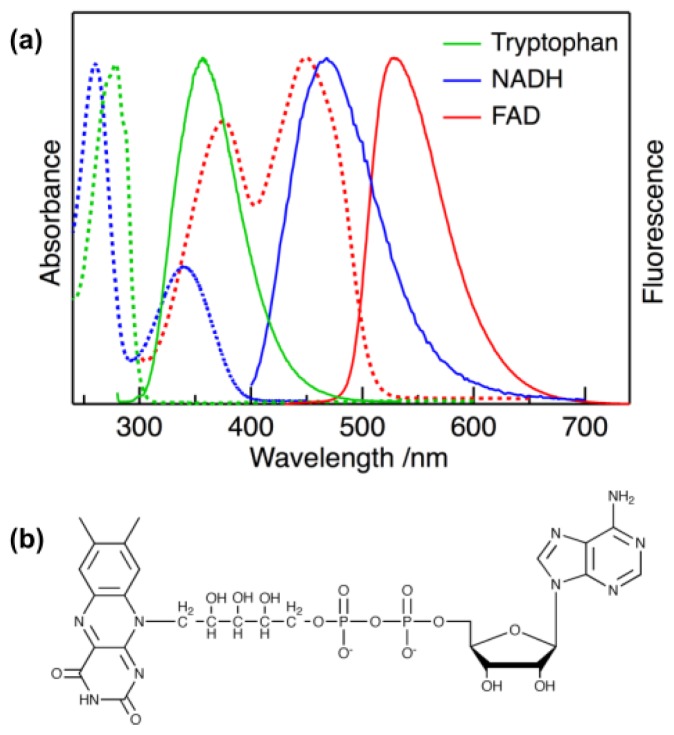
(**a**) Absorption (dotted lines) and fluorescence (solid lines) spectra of representative autofluorescent species in neutral aqueous solution. The maximum intensity is normalized. (**b**) Chemical structure of FAD.

The imaging of fluorescence has been an indispensable tool in cell biology, and numerous fluorescent dyes have been developed for the imaging of living systems at a subcellular level. In recent years, however, the imaging of fluorescence arising from endogenous chromophores in cells and tissues has received much attention because the original cellular environment can be maintained by avoiding the introduction of exogenous probes, and diagnostic tests in medicine can be achieved without staining processes [1–8]. Cells and tissues contain endogenous chromophores exhibiting fluorescence called autofluorescence, and nicotinamide adenine dinucleotide (NADH), flavin adenine dinucleotide (FAD), and amino acids having an aromatic moiety, such as tryptophan, are known as representative autofluorescent chromophores. These chromophores exist in an extensive variety of living systems and are related to cell functions and metabolic activities [2–6]. Absorption and fluorescence spectra of representative autofluorescent chromophores are shown in Figure 1a. Autofluorescent chromophores exhibit characteristic absorption and fluorescence spectra, and each chromophore can be therefore distinguished from others in imaging experiments when excitation and detection wavelengths are properly selected.

We have constructed a fluorescence lifetime imaging (FLIM) system to estimate the microenvironment in a single cell [9–14]. The superior feature of FLIM is that fluorescence lifetime of a chromophore remains unchanged with photobleaching and alternations of optical conditions [15–18]. This is advantageous for quantitative space-resolved estimation of ion concentrations and cellular environment. Intracellular pH is one of the most important factors for understanding cellular conditions, and imaging of intracellular pH using FLIM of exogenous fluorescent dyes or proteins has been performed by several groups [9,10,19–21]. In a previous study, however, we showed that autofluorescence lifetime of NADH in the reduced form, that is one of the representative autofluorescent chromophores, in human cervical carcinoma (HeLa) cells became shorter with increasing intracellular pH, indicating that the pH in a cell can be quantitatively evaluated by FLIM of NADH without exogenous probes [11]. It was also found that the fluorescence lifetime of NADH was not uniform inside a cell and the fluorescence lifetime was shorter in nuclei than in other areas at each pH, and the magnitude of the pH-induced lifetime change was larger in nuclei than in other areas [11]. Then it was proposed that autofluorescence lifetime imaging has a potential to measure a spatial distribution of hydrogen ion concentrations in a cell without dye labeling.

NADH in the reduced form has strong absorption around 340 nm and emits fluorescence in a blue region (Figure 1a), and photoexcitation of NADH in FLIM can be performed by UV light irradiation. When prolonged irradiation is done with UV light, however, living systems may be damaged. If a visible light source can be used, autofluorescence lifetime imaging can become a powerful method applicable to a variety of biological systems. Furthermore, it is difficult in some cases to judge whether the observed lifetime change arises from a change in intracellular pH or a change in other cellular environment affecting the fluorescence lifetime. Therefore, it is very valuable that a change in intracellular pH can also be confirmed by other endogenous chromophores. It is inevitable to examine the intracellular pH dependence of the fluorescence lifetime for different endogenous chromophores.

In the present study, the effect of pH on the fluorescence lifetime of FAD that is excited by visible light, as shown in Figure 1a, was examined in buffer solution and in HeLa cells. We also measured FLIM of FAD in HeLa cells with varying intracellular pH and discussed the possibility to evaluate intracellular pH by measuring the fluorescence lifetime of FAD with excitation of visible light. Both NADH and FAD are important cofactors for energy metabolism and mostly exist as protein-bound state in cells. Therefore comparison of pH dependence of these autofluorescence will lead to an understanding of the mechanism of change in autofluorescence lifetime with different hydrogen ion concentrations.

The chemical structure of FAD is shown in Figure 1b. The isoalloxazine moiety of FAD exhibits absorption and fluorescence in the visible region (Figure 1a). FAD exists mostly as a component of flavoproteins in living systems [22,23] and most flavoproteins are known to show very weak fluorescence due to efficient fluorescence quenching of photoexcited flavin chromophore by surrounding protein environment [24,25]. Only some flavoproteins, including FAD, such as lipoamide dehydrogenase (LipDH) are known to exhibit autofluorescence in cultured cells [26–30]. FAD in neutral aqueous solution exists in two conformations: a non-fluorescent stacked conformation, in which the isoalloxazine and adenine aromatic rings are in close proximity, and a fluorescent open conformation, in which the two aromatic rings are separated from each other [31]. These two conformations can be distinguished from each other by the fluorescence lifetime; the fluorescence lifetime of the stacked conformation is several picoseconds and that of the open conformation is 2–3 ns [32–38]. The short fluorescence lifetime of the stacked conformation comes from fast intramolecular electron transfer between the photoexcited isoalloxazine moiety and the adenine moiety. The two conformations have the same peak wavelengths in absorption and fluorescence spectra, indicating that only the fluorescence lifetime is sensitive to the environment surrounding the isoalloxazine chromophore. We have recently investigated the photoexcitation dynamics of FAD using time-resolved fluorescence techniques and found that the fluorescence lifetimes of both the stacked and open conformations depend on the polar environment surrounding FAD [36].

## 2. Results and Discussion

**Figure 2:**
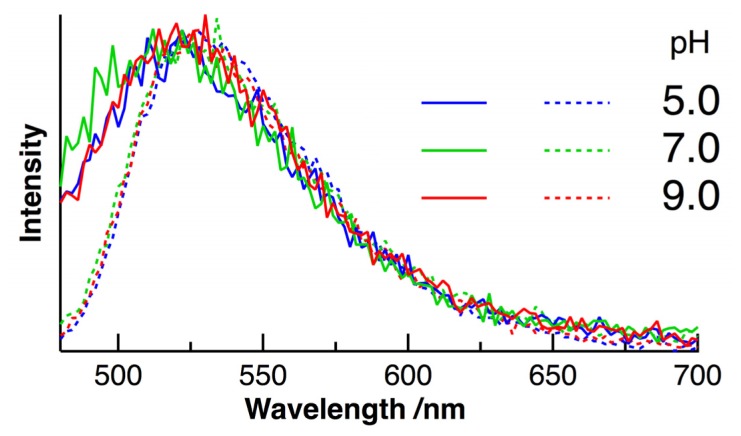
Fluorescence spectra of FAD in HeLa cells (solid lines) and in aqueous buffer solution (dotted lines) at the different pHs of 5.0 (blue), 7.0 (green), and 9.0 (red) following excitation at 450 nm. The maximum intensity is normalized.

Static autofluorescence spectra of HeLa cells with intracellular pH of 5.0, 7.0, and 9.0 are shown in Figure 2. The excitation wavelength was selected to be 450 nm, which effectively excited flavoproteins in cells (see Figure 1a). Cultured HeLa cells were gathered into a quartz cuvette and the autofluorescence spectra of a population of HeLa cells were measured by the time-correlated single photon-counting (TCSPC) method. The number of a population of HeLa cells was estimated to be 10^4^–10^8^ [11]. Some ionophores have been used for *in situ* calibration of the pH in cells, and the present pH dependence experiments for HeLa cells was performed by the so-called nigericin/high K^+^ method [11,21,39–41], in which nigericin (a kind of K^+^/H^+^ polyether ionophore) was added to media to equalize the intracellular pH and extracellular pH. Fluorescence spectra of FAD in aqueous buffer solution at different pH are shown for comparison. The maximum intensity was normalized in all the spectra.

**Figure 3:**
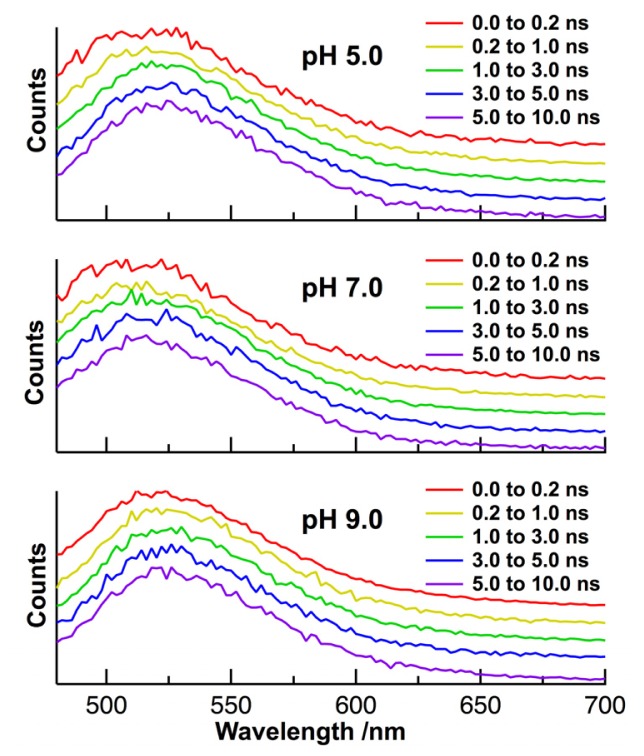
Representative normalized time-resolved autofluorescence spectra of FAD in HeLa cells at different intracellular pHs of 5.0, 7.0, and 9.0. Excitation wavelength was 450 nm.

The absorption band of FAD, with a peak at 450 nm, is assigned to the π–π transition of the isoalloxazine moiety of FAD (Figure 1a). The broad fluorescence of FAD with a peak in the region of 500–600 nm also comes from the isoalloxazine. It is shown in Figure 2 that the shape of the fluorescence spectrum in buffer solution remains unchanged with pHs of 5–9. The intensity of the fluorescence is also independent of aqueous pH, and the shape of the absorption spectrum remains unchanged with pHs of 5–9 (data is not shown). The shape of the fluorescence spectrum in HeLa cells is also unchanged with intracellular pHs of 5–9. The peak of the fluorescence spectrum in HeLa cells is almost the same as that in buffer solution, although a slight broadening in a shorter wavelength region is observed in HeLa cells. FAD serves as a coenzyme in living systems and is regarded to exist in two forms, referred to as protein-bound and free FAD. Thus, it may be said that interactions between FAD and a protein do not significantly affect the peak position of the fluorescence spectrum of FAD. Figure 3 shows intracellular pH dependence of the normalized time-resolved fluorescence spectra of FAD in HeLa cells. The time dependence of the autofluorescence is independent of intracellular pH. Irrespective of pH, the normalized spectrum within the first few nanoseconds shows a slightly higher intensity in the shorter wavelength region of 480–500 nm than the spectra in other time regions. The similar time dependence of the autofluorescence spectra was also reported for FAD in isolated cardiomyocytes [30], and the slight red shift of the fluorescence spectrum at the later stage of time may arise from the so-called dynamics Stokes shift due to solvation [42].

**Figure 4:**
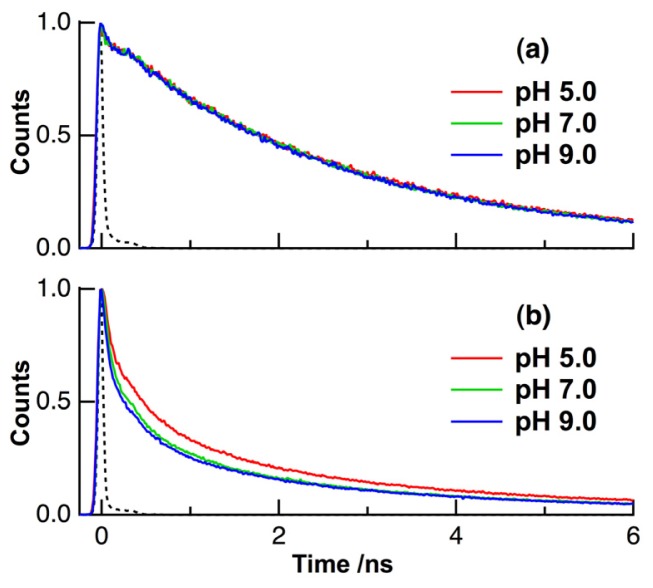
Representative fluorescence decay profiles of FAD in aqueous buffer solution (**a**) and in HeLa cells (**b**) at different pHs of 5.0, 7.0 and 9.0. The intensities are plotted on a linear scale. The instrumental response function is shown in each panel by a dotted line. Excitation and detection wavelengths were 450 and 530 nm, respectively. The maximum intensity at each decay curve is normalized. The total photon number at each decay profile was in the range of 7 × 10^5^–1 × 10^6^.

Fluorescence decay profiles of FAD in aqueous buffer solution at different pHs are shown in Figure 4a. The decay profiles were measured by the TCSPC method. The excitation and fluorescence wavelengths were 450 and 530 nm, respectively. In all the decay profiles, the maximum intensity was normalized to unity. The shape of the decay profile in buffer solution remains unchanged with pHs of 5–9, which is consistent with previous observations [37,38]. The fluorescence of FAD in buffer solution exhibited a multi-exponential decay, which could be fitted with the convolution of the instrumental response function (IRF) by assuming a tetra-exponential decay, *i.e.*, ∑*_i_A_i_*exp(−*t*/τ*_i_*), where *A_i_* and τ*_i_* are the pre-exponential factor and the fluorescence lifetime of component (*i*), respectively [36–38]. The lifetime and pre-exponential factor of these components are shown in Table 1. The fast decaying component (1) is assigned to the stacked conformation of FAD exhibiting the efficient intramolecular electron transfer. Since the lifetime of the component (1) was shorter than the time-width of the present IRF, the τ_1_ value was assumed in the present analysis to be 7 ps, which was obtained by femtosecond time-resolved fluorescence measurements [36]. The component (2) is assigned to the stacked conformation having weak intramolecular interaction between the isoalloxazine and adenine aromatic rings [36,38]. The components (3) and (4), which have the nanosecond fluorescence lifetime, are assigned to the open conformation of FAD [36–38]. All the fitted values remained constant with pHs of 5–9 in buffer solution.

The measurements of fluorescence decay profiles of FAD in a population of HeLa cells were also carried out with intracellular pH of 5.0, 7.0 and 9.0 (Figure 4b). Cultured HeLa cells were gathered into a quartz cuvette, and the nigericin/high K^+^ method was used for the calibration of intracellular pH, as mentioned above. The decay profile slightly depended on the sample cells, and the average of decay profiles for four different samples was shown in Figure 4b. The decay profile in HeLa cells is different from that of in buffer solution, and the picosecond-lifetime component is markedly observed in HeLa cells. It is also shown in Figure 4b that the decay constant in HeLa cells becomes larger with increasing intracellular pH. The decay profiles of FAD in HeLa cells were fitted by assuming a tetra-exponential decay, and the obtained lifetime and pre-exponential factor at each pH are shown in Table 1.

In Table 1, the two picosecond-lifetime components, having lifetimes of ~80 ps and 700 ps, *i.e.*, components (1) and (2), respectively, are obtained from HeLa cells. The sum of the pre-exponential factors of the components (1) and (2) is larger than 0.7, indicating that the picosecond-lifetime components become dominant in HeLa cells. The decaying components with lifetimes of hundreds of picoseconds were observed for autofluorescence of FAD in isolated cardiomyocytes [30]. It was also reported that the fluorescence of LipDH, which is a FAD-containing protein, exhibited a multi-exponential decay with subnanosecond and nanosecond fluorescence lifetimes [30,43]. Since FAD exists as protein-bound and free species in living systems, the picosecond-lifetime components (1) and (2) having lifetime of 80–700 ps can be assigned to FAD bound in enzymes such as LipDH. It is difficult in the present study to judge whether the component (3) having a nanosecond fluorescence lifetime is assigned to the open conformation of free FAD or the nanosecond lifetime component of FAD-containing proteins. It was necessary to consider the component having a lifetime of ~10 ns to reproduce the decay profile in a longer time range, although its pre-exponential factor was 2%–3%.

The average fluorescence lifetime (τ_ave_) is given by ∑*A*_i_τ_i_ (*i* = 1–4) in Table 1. The τ_ave_ values of FAD in HeLa cells were evaluated to be 1.11 ns, 0.84 ns, and 0.78 ns for intracellular pHs of 5.0, 7.0, and 9.0, respectively. Thus, τ_ave_ of FAD in HeLa cells decreases with increasing intracellular pH, while the shape of the fluorescence spectrum remains unchanged with pH. By using such a pH dependence of τ_ave_ of FAD in living cells, the intracellular pH of a cell can be evaluated without dye labeling. As shown in Table 1, the pre-exponential factor of the component (1) ascribed to protein-bound FAD changed significantly with different intracellular pHs, resulting in the reduction of τ_ave_ with the increase of intracellular pH. It is therefore concluded that the observed pH dependence of τ_ave_ in HeLa cells comes from the pH dependence of interactions between FAD and the enzyme to which it is bound. It should be noted that the fluorescence decay of isolated FAD in aqueous solution remained unchanged with different pHs (see Figure 4a), which is consistent with the present conclusion. Femtosecond time-resolved measurements may be useful to detect interactions between FAD and a protein because electron transfer between FAD and a protein will occur in the femtosecond to picosecond time scale, and the rate of electron transfer depends on donor-acceptor distance [24,25].

**Figure 5:**
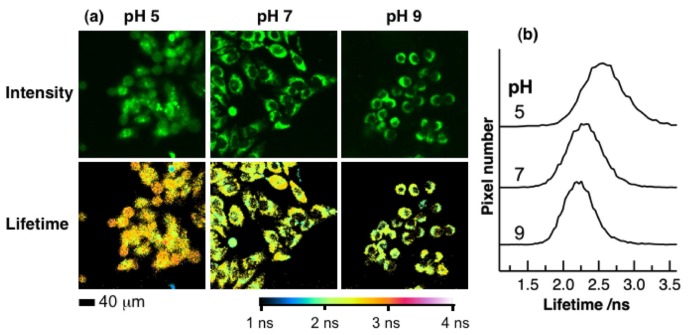
(**a**) Autofluorescence intensity images (upper) and corresponding lifetime images (lower) of HeLa cells at intracellular pH indicated at the top. Scale bar given in the bottom is 40 μm. (**b**) Intracellular pH dependence of the histogram of the fluorescence lifetime obtained from the whole area of the fluorescence lifetime image in (**a**). Excitation and monitoring wavelengths were 450 and 515–560 nm, respectively.

We have also measured fluorescence lifetime images of FAD in HeLa cells at different intracellular pHs by the time-gated detection method with four time-windows [9–14]. The obtained autofluorescence lifetime and corresponding intensity images are shown in Figure 5a. Excitation wavelength was 450 nm, and autofluorescence in the region of 515–560 nm was detected. To minimize the amount of data generated, the fluorescence lifetime at each pixel of the image was evaluated by analyzing the four time-gated signals by assuming a single exponential decay. The intensity of autofluorescence reflects the concentration of FAD in organelle, and the dull round region in the intensity image of a cell is assigned to a nucleus. The pseudocolor of the fluorescence lifetime image depends on intracellular pH, and the marked difference is observed between the images at intracellular pHs of 5 and 7. The pH dependence of the normalized histogram of the fluorescence lifetime obtained from the whole area of the image is shown in Figure 5b. The lifetime at the peak of the histogram was larger at an intracellular pH of 5 than those at an intracellular pH of 7 and 9, in agreement with the result of the fluorescence decays shown in Figure 4b. Reproducibility was also checked using more than six different cultured cells and a different stock of cells, and it was confirmed that the observed behavior was not related to cell death. The lifetimes at the peak of the histogram at intracellular pH of 5, 7, and 9 were 2.53, 2.32, and 2.22 ns, respectively. The uncertainty of the peak position was ~±10%. It should be noted that the lifetime obtained by the present time-gated FLIM is longer than that evaluated by the fluorescence decay profile with TCSPC (Table 1) at each pH, which is due to the fact that a part of the fast decaying portion following photoexcitation was cut in the time-gated detection method to exclude scattered excitation light, and the signal integration was made from ~1 ns after photoexcitation [11].

## 3. Experimental Section

### 3.1. Sample Preparation

HeLa cells were grown in Dulbecco’s modified Eagle’s medium (DMEM) supplemented with 10% fetal bovine serum, 1 × 10^5^ U/L penicillin G and 100 mg/L streptomycin sulfate in a 5% CO_2_ humidified atmosphere at 37 °C for one to two days. Calibration of intracellular pH was performed by the addition of nigericin to culture media. Nigericin equilibrates proton concentration across the plasma membrane in the presence of a depolarizing extracellular concentration of K^+^ [11,21,39–41]. In the imaging measurements, HeLa cells grown on LAB-TEK 8-well chambered coverslips (Nalge Nunc International) were rinsed twice with KCl-rich media (125 mM KCl, 10 mM HEPES, 10 mM MES, 0.5 mM CaCl_2_, 0.5 mM MgCl_2_, 30 μM nigericin) and then incubated with the same KCl-rich media at different pHs. In the bulk measurements, HeLa cells prepared with KCl-rich media (125 mM KCl, 10 mM HEPES, 10 mM MES, 0.5 mM CaCl_2_, 0.5 mM MgCl_2_, 0.1 mM nigericin) at different pHs were gathered into a quartz cuvette with a 2 mm optical path [11] and then incubated with the same media. The medium pH was adjusted using HCl and NaOH solutions.

### 3.2. Time-Resolved Fluorescence Measurements

Fluorescence lifetime images were measured using a four-channel time-gated detection system [9–14]. The second harmonic of a mode-locked Ti:sapphire laser (Spectra Physics) pumped by a Millennia Xs diode laser (Spectra Physics, Santa Clara, CA, USA) was used for excitation. The pulse duration and the repetition rate of the laser pulse were 80 fs and 81 MHz, respectively. The excitation beam was introduced into a scanner head (Nikon, Tokyo, Japan) of an inverted confocal microscope (Nikon). The excitation beam was focused onto HeLa cells placed on the stage of the microscope through a 40× objective lens (Nikon, CFI S Fluor, NA 0.90), and the fluorescence from the cells was collected with the same objective lens and transmitted into the scanner head, followed by interference filters (Nikon) to select detection wavelength of the fluorescence. The fluorescence was detected by a photomultiplier in a high-speed lifetime imaging module (Nikon Europe BV). The fluorescence decay profile was measured for each pixel of the image. The detected fluorescence photons were accumulated in one of the four time-windows set by the lifetime imaging module. The fluorescence lifetime of each pixel of the image was evaluated from the analysis of the four time-window signals by assuming a single exponential decay, and the fluorescence lifetime image was obtained. The background was evaluated by counts at the area where cells were not observed. The size of the image was 256 × 256 pixels. All of the time windows were set to be 2.0 ns.

A home-made TCSPC system was used both for the bulk measurements of fluorescence decay profiles and for the measurements of time-resolved fluorescence spectra of FAD in HeLa cells and in aqueous solution [11]. The second harmonic of the mode-locked Ti:sapphire laser was used for excitation, and its repetition rate was reduced to ~6 MHz using an electro-optic modulator (Conoptics, Danbury, CT, USA). Fluorescence from the sample was dispersed by a monochromator (Nikon) and then detected by a microchannel-plate photomultiplier (MCP-PMT, Hamamatsu, Hamamatsu, Japan). Fluorescence signal was amplified, discriminated, and then led to a time-to-amplitude converter system as a start pulse. Fluorescence decays were obtained with a multichannel pulse height analyzer (SEIKO EG & G, Tokyo, Japan). The spectral width of the monochromator was ~5 nm. The IRF had a full width at half maximum (FWHM) of ~60 ps. Fluorescence decays were simulated with the convolution of the IRF with a multi-exponential decay function. The lifetime longer than ~10 ps can be evaluated using the convolution method under the present experimental conditions [44]. In the measurements of time-resolved fluorescence spectra, a series of fluorescence decays were measured as a function of fluorescence wavelength with a step of 2 nm, and time-resolved fluorescence spectra having different time windows were obtained.

## 4. Conclusions

Autofluorescence spectrum and autofluorescence lifetime of endogenous FAD in HeLa cells were measured with different intracellular pHs. The shape of the autofluorescence spectrum remains unchanged with intracellular pHs of 5–9; however, the average autofluorescence lifetime of FAD becomes shorter as the intracellular pH increases, suggesting that intracellular pH of a cell can be evaluated using autofluorescence lifetime of endogenous FAD without exogenous dyes. Picosecond-lifetime components assigned to protein-bound FAD are observed in autofluorescence of FAD in HeLa cells. The pre-exponential factor of the fast decaying component with a lifetime of tens of picoseconds increases as intracellular pH increases, resulting in the reduction of the average autofluorescence lifetime with the increase of intracellular pH. This result indicates that interactions between FAD and surrounding functional groups in a protein depend on intracellular pH, which causes the pH-induced change in the non-radiative decay rate of FAD in HeLa cells. This conclusion is consistent with the fact that the fluorescence lifetime of isolated FAD in buffer solution remains unchanged with pHs of 5 to 9. We believe that autofluorescence lifetime imaging can be applied to measure changes in intracellular pH with physiological processes such as apoptosis [45].

## Figures and Tables

**Table 1:** Fitting parameters of fluorescence decay profiles of FAD in aqueous buffer solution and in HeLa cells at different pH a,b.

	pH	τ_1_ (ps)	τ_2_ (ps)	τ_3_ (ns)	τ_4_ (ns)	τ_ave_ (ns)
solution	5, 7, 9	7 (0.66) c	220 (0.03)	2.09 (0.17)	3.97 (0.14)	2.70 d
HeLa cells	5	80 (0.52)	700 (0.27)	3.17 (0.18)	10.2 (0.03)	1.11
	7	80 (0.60)	700 (0.25)	3.17 (0.13)	10.2 (0.02)	0.84
	9	80 (0.63)	700 (0.23)	3.17 (0.12)	9.42 (0.02)	0.78

a Pre-exponential factor of each component is given in parenthesis.

b Experimental errors of τ_1_, τ_2_, τ_3_ and τ_4_ are ca. ±30, ±20, ±10 and ±30%, respectively.

c τ_1_ in solution was fixed to be 7 ps.

d τ_1_ was not included in the calculation of τ_ave_ in solution.

## References

[1] Huang S., Heikal A.A., Webb W.W. (2002). Two-photon fluorescence spectroscopy and microscopy of NAD(P)H and flavoprotein. Biophys. J.

[2] Niesner R., Peker B., Schlüsche P., Gericke K.-H. (2004). Noniterative biexponential fluorescence lifetime imaging in the investigation of cellular metabolism by means of NAD(P)H autofluorescence. ChemPhysChem.

[3] Skala M.C., Riching K.M., Gendron-Fitzpatrick A., Eickhoff J., Eliceiri K.W., White J.G., Ramanujam N. (2007). *In vivo* multiphoton microscopy of NADH and FAD redox states, fluorescence lifetimes, and cellular morphology in precancerous epithelia. Proc. Natl. Acad. Sci. USA.

[4] Schweitzer D., Schenke S., Hammer M., Schweitzer F., Jentsch S., Birckner E., Becker W., Bergmann A. (2007). Towards metabolic mapping of the human retina. Microsc. Res. Tech.

[5] Yu Q., Heikal A.A. (2009). Two-photon autofluorescence dynamics imaging reveals sensitivity of intracellular NADH concentration and conformation to cell physiology at the single-cell level. J. Photochem. Photobiol. B.

[6] Ghukasyan V.V., Kao F.J. (2009). Monitoring cellular metabolism with fluorescence lifetime of reduced nicotinamide adenine dinucleotide. J. Phys. Chem. C.

[7] Chorvat D., Chorvatova A. (2009). Multi-wavelength fluorescence lifetime spectroscopy: A new approach to the study of endogenous fluorescence in living cells and tissues. Laser Phys. Lett..

[8] Berezin M.Y., Achilefu S. (2010). Fluorescence lifetime measurements and biological imaging. Chem. Rev.

[9] Wang H.-P., Nakabayashi T., Tsujimoto K., Miyauchi S., Kamo N., Ohta N. (2007). Fluorescence lifetime image of a single halobacterium. Chem. Phys. Lett.

[10] Nakabayashi T., Wang H.-P., Kinjo M., Ohta N. (2008). Application of fluorescence lifetime imaging of enhanced green fluorescent protein to intracellular pH measurements. Photochem. Photobiol. Sci.

[11] Ogikubo S., Nakabayashi T., Adachi T., Islam M.S., Yoshizawa T., Kinjo M., Ohta N. (2011). Intracellular pH sensing using autofluorescence lifetime microscopy. J. Phys. Chem. B.

[12] Nakabayashi T., Nagao I., Kinjo M., Aoki Y., Tanaka M., Ohta N. (2008). Stress-induced environmental changes in a single cell as revealed by fluorescence lifetime imaging. Photochem. Photobiol. Sci.

[13] Ito T., Oshita S., Nakabayashi T., Sun F., Kinjo M., Ohta N. (2009). Fluorescence lifetime images of green fluorescent protein in HeLa cells during TNF-alpha induced apoptosis. Photochem. Photobiol. Sci.

[14] Awasthi K., Nakabayashi T., Ohta N. (2012). Application of nanosecond pulsed electric fields into HeLa cells expressing enhanced green fluorescent protein and fluorescence lifetime microscopy. J. Phys. Chem. B.

[15] Wallrabe H., Periasamy A. (2005). Imaging protein molecules using FRET and FLIM microscopy. Curr. Opin. Biotechnol.

[16] Becker W., Bergmann A., Biskup C. (2007). Multispectral fluorescence lifetime imaging by TCSPC. Microsc. Res. Tech.

[17] Levitt J.A., Matthews D.R., Ameer-Beg S.M., Suhling K. (2009). Fluorescence lifetime and polarization-resolved imaging in cell biology. Curr. Opin. Biotechnol.

[18] Borst J.W., Visser A.J.W.G. (2010). Fluorescence lifetime imaging microscopy in life sciences. Meas. Sci. Technol.

[19] Hanson K.M., Behne M.J., Barry N.P., Mauro T.M., Gratton E., Clegg R.M. (2002). Two-photon fluorescence lifetime imaging of the skin stratum corneum pH gradient. Biophys. J.

[20] Niesner R., Peker B., Schlüsche P., Gericke K.-H., Hoffmann C., Hahne D., Müller-Goymann C. (2005). 3D-resolved investigation of the pH gradient in artificial skin constructs by means of fluorescence lifetime imaging. Pharmaceu. Res.

[21] Lin H.-J., Herman P., Lakowicz J.R. (2003). Fluorescence lifetime-resolved pH imaging of living cells. Cytometry.

[22] Ghisla S., Massey V. (1989). Mechanisms of flavoprotein-catalyzed reactions. Eur. J. Biochem.

[23] Mattevi A. (2006). To be or not to be an oxidase: Challenging the oxygen reactivity of flavoenzymes. Trends Biochem. Sci.

[24] Mataga N., Chosrowjan H., Shibata Y., Tanaka F., Nishima Y., Shiga K. (2000). Dynamics and mechanisms of ultrafast fluorescence quenching reactions of flavin chromophores in protein nanospace. J. Phys. Chem. B.

[25] Chosrowjan H., Taniguchi S., Mataga N., Nakanishi T., Haruyama Y., Sato S., Kitamura M., Tanaka F. (2010). Effects of the disappearance of one charge on ultrafast fluorescence dynamics of the FMN binding protein. J. Phys. Chem. B.

[26] Hall C.L., Kamin H. (1975). The purification and some properties of electron transfer flavoprotein and general fatty acyl coenzyme A dehydrogenase from pig liver mitochondria. J. Biol. Chem.

[27] Kunz W.S., Kunz W. (1985). Contribution of different enzymes to flavoprotein fluorescence of isolated rat liver mitochondria. Biochim. Biophys. Acta.

[28] Kunz W.S. (1986). Spectral properties of fluorescent flavoproteins of isolated rat liver mitochondria. FEBS Lett.

[29] Romashko D.N., Marban E., O’Rourke B. (1998). Subcellular metabolic transients and mitochondrial redox waves in heart cells. Proc. Natl. Acad. Sci. USA.

[30] Chorvat D., Chorvatova A. (2006). Spectrally resolved time-correlated single photon counting: A novel approach for characterization of endogenous fluorescence in isolated cardiac myocytes. Eur. Biophys. J..

[31] Barrio J.R., Tolman G.L., Leonard N.J., Spencer R.D., Weber G. (1973). Flavin 1, *N*^6^-ethenoadenine dinucleotide: Dynamic and static quenching of fluorescence. Proc. Natl. Acad. Sci. USA.

[32] Stanley R.J., MacFarlane A.W. (2000). Ultrafast excited state dynamics of oxidized flavins: Direct observations of quenching by purines. J. Phys. Chem. A.

[33] Van den Berg P.A.W., Feenstra K.A., Mark A.E., Berendsen H.J.C., Visser A.J.W.G. (2002). Dynamic conformations of flavin adenine dinucleotide: Simulated molecular dynamics of the flavin cofactor related to the time-resolved fluorescence characteristics. J. Phys. Chem. B.

[34] Kao Y.-T., Saxena C., He T.-F., Guo L., Wang L., Sancar A., Zhong D. (2008). Ultrafast dynamics of flavins in five redox states. J. Am. Chem. Soc.

[35] Li G., Glusac K.D. (2008). Light-triggered proton and electron transfer in flavin cofactors. J. Phys. Chem. A.

[36] Nakabayashi T., Islam M.S., Ohta N. (2010). Fluorescence decay dynamics of flavin adenine dinucleotide in a mixture of alcohol and water in the femtosecond and nanosecond time range. J. Phys. Chem. B.

[37] Islam S.D.M., Susdorf T., Penzkofer A., Hegemann P. (2003). Fluorescence quenching of flavin adenine dinucleotide in aqueous solution by pH dependent isomerisation and photo-induced electron transfer. Chem. Phys.

[38] Sengupta A., Khade R.V., Hazra P. (2011). pH dependent dynamic behavior of flavin mononucleotide (FMN) and flavin adenine dinucleotide (FAD) in femtosecond to nanosecond time scale. J. Photochem. Photobiol. A.

[39] Thomas J.A., Buchsbaum R.N., Zimniak A., Racker E. (1979). Intracellular pH measurements in ehrlich ascites tumor cells utilizing spectroscopic probes generated in situ. Biochemistry.

[40] Nedergaard M., Desai S., Pulsinelli W. (1990). Dicarboxy-dichlorofluorescein: A new fluorescent probe for measuring acidic intracellular pH. Anal. Biochem.

[41] Sanders R., Draaijer A., Gerritsen H.C., Houpt P.M., Levine Y.K. (1995). Quantitative pH imaging in cells using confocal fluorescence lifetime imaging microscopy. Anal. Biochem.

[42] Gardecki J.A., Maroncelli M. (1999). Comparison of the single-wavelength and spectral-reconstruction methods for determining the solvation-response function. J. Phys. Chem. A.

[43] Bastiaens P.I.H., van Hoek A., Wolkers W.F., Brochon J.-C., Visser A.J.W.G. (1992). Comparison of the dynamical structures of lipoamide dehydrogenase and glutathione reductase by time-resolved polarized flavin fluorescence. Biochemistry.

[44] Yamazaki I., Tamai N., Kume H., Tsuchiya H., Oba K. (1985). Microchannel-plate photomultiplier applicability to the time-correlated photon-counting method. Rev. Sci. Instrum.

[45] Matsuyama S., Llopis J., Deveraux Q.L., Tsien R.Y., Reed J.C. (2000). Changes in intramitochondrial and cytosolic pH: Early events that modulate caspase activation during apoptosis. Nat. Cell Biol..

